# Tetra­kis(pyridazine-κ*N*)bis­(seleno­cyanato-κ*N*)nickel(II) pyridazine disolvate

**DOI:** 10.1107/S1600536812028036

**Published:** 2012-06-23

**Authors:** Susanne Wöhlert, Mario Wriedt, Inke Jess, Christian Näther

**Affiliations:** a Institut für Anorganische Chemie, Christian-Albrechts-Universität Kiel, Max-Eyth-Strasse 2, 24118 Kiel, Germany; b Departement of Chemistry, Texas A&M University, College Station, Texas 77843, USA

## Abstract

The reaction of nickel(II) nitrate with potassium seleno­cyanate and pyridazine leads to crystals of the title compound, [Ni(NCSe)_2_(C_4_H_4_N_2_)_4_]·2C_4_H_4_N_2_. The Ni^II^ atom is coordinated by two terminal *N*-bonded seleno­cyanate anions and four pyridazine ligands within a slightly distorted octa­hedral geometry. The crystal structure contains two crystallographically independent pyridazine molecules in cavities of the structure, which are not coordinated to the metal centres. The structure is pseudo-*C*-centered due to the positioning of the discrete coordination complexes; the non-coordinating pyridazine molecules, however, break the *C*-centering. In the subcell, these ligands are disordered around centres of inversion, which do not coincide with the mid-point of the mol­ecules.

## Related literature

For the synthesis, structures and properties of related coordination compounds see: Boeckmann & Näther (2010 ▶, 2011 ▶); Wöhlert *et al.* (2011 ▶, 2012 ▶).
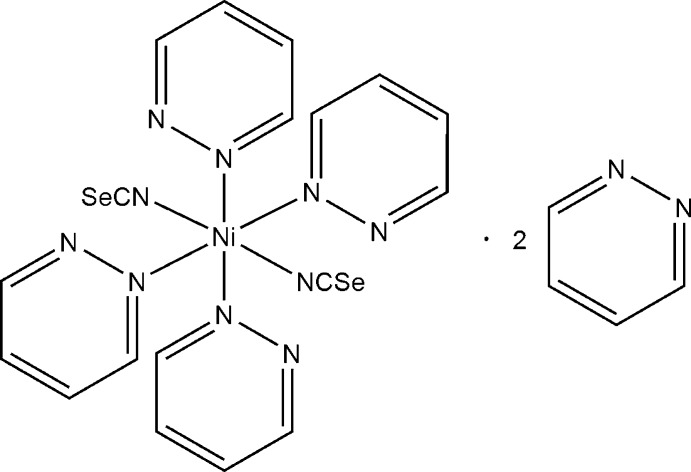


## Experimental

### 

#### Crystal data

[Ni(NCSe)_2_(C_4_H_4_N_2_)_4_]·2C_4_H_4_N_2_
*M* *_r_* = 749.22
Triclinic, 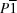

*a* = 11.2923 (15) Å
*b* = 12.0868 (14) Å
*c* = 12.8220 (15) Å
α = 62.324 (9)°
β = 88.427 (10)°
γ = 88.512 (10)°
*V* = 1549.1 (3) Å^3^
*Z* = 2
Mo *K*α radiation
μ = 3.02 mm^−1^
*T* = 293 K
0.25 × 0.14 × 0.10 mm

#### Data collection

Stoe IPDS-2 diffractometer
Absorption correction: numerical (*X-SHAPE* and *X-RED32*; Stoe & Cie, 2008 ▶) *T*_min_ = 0.243, *T*_max_ = 0.572
15038 measured reflections
6534 independent reflections
4010 reflections with *I* > 2σ(*I*)
*R*_int_ = 0.068

#### Refinement

*R*[*F* ^2^ > 2σ(*F* ^2^)] = 0.053
*wR*(*F* ^2^) = 0.129
*S* = 1.01
6534 reflections
388 parameters
H-atom parameters constrained
Δρ_max_ = 0.57 e Å^−3^
Δρ_min_ = −0.46 e Å^−3^

### 

Data collection: *X-AREA* (Stoe & Cie, 2008 ▶); cell refinement: *X-AREA*; data reduction: *X-AREA*; program(s) used to solve structure: *SHELXS97* (Sheldrick, 2008 ▶); program(s) used to refine structure: *SHELXL97* (Sheldrick, 2008 ▶); molecular graphics: *SHELXTL* (Sheldrick, 2008 ▶) and *DIAMOND* (Brandenburg, 2011 ▶); software used to prepare material for publication: *XCIF* in *SHELXTL* and *PLATON* (Spek, 2009 ▶).

## Supplementary Material

Crystal structure: contains datablock(s) I, global. DOI: 10.1107/S1600536812028036/vn2044sup1.cif

Structure factors: contains datablock(s) I. DOI: 10.1107/S1600536812028036/vn2044Isup2.hkl

Additional supplementary materials:  crystallographic information; 3D view; checkCIF report

## Figures and Tables

**Table 1:** Selected bond lengths (Å)

Ni1—N1	2.051 (4)
Ni1—N2	2.055 (4)
Ni1—N10	2.154 (3)
Ni1—N20	2.129 (3)
Ni1—N30	2.153 (3)
Ni1—N40	2.124 (3)

**Fig. 1.**
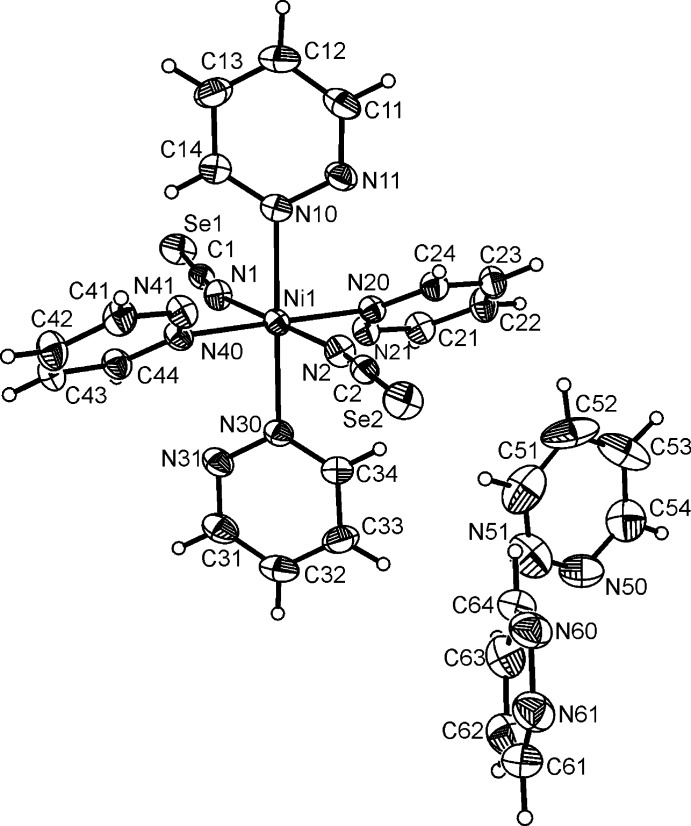
Crystal structure of the title compound [Ni(NCSe)2(C4H4N2)4].2C4H4N2 with labeling and displacement ellipsoids drawn at the 30% probability level.

## supplementary crystallographic information

### Comment

Recently we have reported on the synthesis and characterization of coordination polymers based on transition metal thio- and selenocyanates. Within this project we investigated the influence of neutral N-donor co-ligands on the structural, thermal and magnetic properties of compounds, in which the metal cations are linked by the anionic ligands (Boeckmann & Näther, 2010, 2011; Wöhlert *et al.*, 2011). In the present investigation we tried to prepare similar compounds using pyridazine as co-ligand, which results in the formation of single crystals of the title compound, which are isotypic to [Ni(NCS)_2_(pyridazine)_4_].2(pyridazine) reported recently (Wöhlert *et al.*, 2012). In the crystal structure each nickel(II) cation is coordinated by two terminal N-bonded selenocyanato anions and four pyridazine ligands into discrete complexes (Fig. 1). The NiN_6_ octahedra are slightly distorted with distances ranging from 2.051 (4) to 2.154 (3) Å and angles between 87.31 (12) ° and 179.88 (15) ° (Table 1). The discrete complexes are arranged in layers, which are separated by additional non-coordinated pyridazine ligands. The shortest intermolecular Ni···Ni distance amounts to 8.2173 (12) Å.

### Experimental

Nickel(II) nitrate hexahydrate (Ni(NO_3_)_2_x6H_2_O) and potassium selenocyanate (KNCSe) as well as pyridazine were obtained from Alfa Aesar. All chemicals were used without further purification. 0.125 mmol (36.4 mg) Ni(NO_3_)_2_.6H_2_O and 0.25 mmol (36.0 mg) KNCSe were reacted in 2.76 mmol (200 µ*L*) pyridazine, respectively. Purple single crystals of the title compound were obtained after one week.

### Refinement

All H atoms were located in a difference map but were positioned with idealized geometry and were refined isotropically with *U*_iso_(H) = 1.2 *U*_eq_(C) of the parent atom using a riding model with C—H = 0.93 Å. *PLATON* (Spek, 2009) detects a pseudo-*C* centreing. In the subcell the two crystallographically independent pyridazine molecules are each located on centres of inversion, which means that at this place the crystal symmetry is higher than the molecular symmetry. Moreover, the midpoint of these rings does not coincidence with the inversion centre and therefore, after generating the symmetry equivalent atoms two different orientations of the pyridazine rings are obtained, which cannot be resolved successfully. After structure refinement all reliability factors are much higher than in the supercell. Therefore, the structure was refined in the supercell in which both non-coordinating pyridazine ligands are perfectly ordered.

### Crystal data

**Table:**

[Ni(NCSe)_2_(C_4_H_4_N_2_)_4_]·2C_4_H_4_N_2_	*Z* = 2
*M**_r_* = 749.22	*F*(000) = 748
Triclinic, *P*1	*D*_x_ = 1.606 Mg m^−^^3^
Hall symbol: -P 1	Mo *K*α radiation, λ = 0.71073 Å
*a* = 11.2923 (15) Å	Cell parameters from 15038 reflections
*b* = 12.0868 (14) Å	θ = 1.8–26.8°
*c* = 12.8220 (15) Å	µ = 3.02 mm^−^^1^
α = 62.324 (9)°	*T* = 293 K
β = 88.427 (10)°	Block, purple
γ = 88.512 (10)°	0.25 × 0.14 × 0.10 mm
*V* = 1549.1 (3) Å^3^	

### Data collection

**Table:**

Stoe IPDS-2 diffractometer	6534 independent reflections
Radiation source: fine-focus sealed tube	4010 reflections with *I* > 2σ(*I*)
Graphite monochromator	*R*_int_ = 0.068
ω scan	θ_max_ = 26.8°, θ_min_ = 1.8°
Absorption correction: numerical (*X-SHAPE* and *X-RED32*; Stoe & Cie, 2008)	*h* = −13→14
*T*_min_ = 0.243, *T*_max_ = 0.572	*k* = −15→15
15038 measured reflections	*l* = −16→16

### Refinement

**Table:**

Refinement on *F*^2^	Primary atom site location: structure-invariant direct methods
Least-squares matrix: full	Secondary atom site location: difference Fourier map
*R*[*F*^2^ > 2σ(*F*^2^)] = 0.053	Hydrogen site location: inferred from neighbouring sites
*wR*(*F*^2^) = 0.129	H-atom parameters constrained
*S* = 1.01	*w* = 1/[σ^2^(*F*_o_^2^) + (0.0583*P*)^2^] where *P* = (*F*_o_^2^ + 2*F*_c_^2^)/3
6534 reflections	(Δ/σ)_max_ = 0.001
388 parameters	Δρ_max_ = 0.57 e Å^−^^3^
0 restraints	Δρ_min_ = −0.46 e Å^−^^3^

### Special details

**Table:**

Geometry. All e.s.d.'s (except the e.s.d. in the dihedral angle between two l.s. planes) are estimated using the full covariance matrix. The cell e.s.d.'s are taken into account individually in the estimation of e.s.d.'s in distances, angles and torsion angles; correlations between e.s.d.'s in cell parameters are only used when they are defined by crystal symmetry. An approximate (isotropic) treatment of cell e.s.d.'s is used for estimating e.s.d.'s involving l.s. planes.
Refinement. Refinement of *F*^2^ against ALL reflections. The weighted *R*-factor *wR* and goodness of fit *S* are based on *F*^2^, conventional *R*-factors *R* are based on *F*, with *F* set to zero for negative *F*^2^. The threshold expression of *F*^2^ > σ(*F*^2^) is used only for calculating *R*-factors(gt) *etc*. and is not relevant to the choice of reflections for refinement. *R*-factors based on *F*^2^ are statistically about twice as large as those based on *F*, and *R*- factors based on ALL data will be even larger.

### Fractional atomic coordinates and isotropic or equivalent isotropic displacement parameters (Å^2^)

**Table:**

	*x*	*y*	*z*	*U*_iso_*/*U*_eq_	
Ni1	0.75263 (4)	0.75195 (4)	0.49428 (5)	0.03775 (13)	
N1	0.6464 (3)	0.8464 (3)	0.3514 (3)	0.0482 (8)	
C1	0.5667 (4)	0.8920 (3)	0.2914 (4)	0.0449 (9)	
Se1	0.44241 (5)	0.96264 (4)	0.19891 (5)	0.06887 (18)	
N2	0.8589 (3)	0.6575 (3)	0.6376 (3)	0.0480 (8)	
C2	0.9339 (4)	0.6062 (3)	0.7026 (4)	0.0433 (9)	
Se2	1.05284 (4)	0.52717 (4)	0.80123 (5)	0.06587 (17)	
N10	0.6978 (3)	0.5804 (3)	0.4987 (3)	0.0455 (8)	
N11	0.6601 (3)	0.4895 (3)	0.6020 (3)	0.0550 (9)	
C11	0.6253 (4)	0.3838 (4)	0.6056 (5)	0.0663 (13)	
H11	0.5970	0.3218	0.6772	0.080*	
C12	0.6283 (4)	0.3596 (4)	0.5112 (5)	0.0687 (14)	
H12	0.6036	0.2835	0.5183	0.082*	
C13	0.6688 (5)	0.4513 (5)	0.4069 (5)	0.0743 (15)	
H13	0.6742	0.4403	0.3398	0.089*	
C14	0.7019 (4)	0.5629 (4)	0.4047 (4)	0.0589 (11)	
H14	0.7279	0.6278	0.3337	0.071*	
N20	0.6117 (3)	0.7459 (3)	0.6104 (3)	0.0408 (7)	
N21	0.5425 (3)	0.8496 (3)	0.5696 (3)	0.0466 (8)	
C21	0.4570 (4)	0.8558 (4)	0.6387 (4)	0.0541 (11)	
H21	0.4098	0.9277	0.6104	0.065*	
C22	0.4334 (4)	0.7615 (4)	0.7510 (4)	0.0623 (12)	
H22	0.3721	0.7692	0.7972	0.075*	
C23	0.5033 (4)	0.6571 (4)	0.7912 (4)	0.0568 (11)	
H23	0.4907	0.5903	0.8655	0.068*	
C24	0.5940 (4)	0.6534 (4)	0.7177 (4)	0.0480 (9)	
H24	0.6441	0.5837	0.7448	0.058*	
N30	0.8051 (3)	0.9236 (3)	0.4909 (3)	0.0456 (8)	
N31	0.8360 (3)	1.0191 (3)	0.3876 (3)	0.0557 (9)	
C31	0.8674 (4)	1.1253 (4)	0.3867 (5)	0.0661 (13)	
H31	0.8906	1.1907	0.3148	0.079*	
C32	0.8680 (4)	1.1452 (5)	0.4824 (6)	0.0712 (14)	
H32	0.8892	1.2216	0.4768	0.085*	
C33	0.8360 (5)	1.0471 (5)	0.5874 (5)	0.0735 (15)	
H33	0.8345	1.0536	0.6569	0.088*	
C34	0.8056 (4)	0.9371 (4)	0.5858 (4)	0.0581 (11)	
H34	0.7845	0.8691	0.6568	0.070*	
N40	0.8959 (3)	0.7555 (3)	0.3819 (3)	0.0438 (7)	
N41	0.9615 (3)	0.6503 (3)	0.4249 (3)	0.0487 (8)	
C41	1.0520 (4)	0.6428 (4)	0.3616 (4)	0.0589 (12)	
H41	1.0956	0.5685	0.3908	0.071*	
C42	1.0859 (4)	0.7394 (5)	0.2542 (5)	0.0652 (13)	
H42	1.1526	0.7319	0.2135	0.078*	
C43	1.0186 (4)	0.8460 (4)	0.2098 (4)	0.0603 (12)	
H43	1.0364	0.9137	0.1373	0.072*	
C44	0.9220 (4)	0.8487 (4)	0.2779 (4)	0.0505 (10)	
H44	0.8735	0.9196	0.2487	0.061*	
N50	0.8139 (5)	0.8781 (4)	0.9912 (5)	0.0856 (14)	
N51	0.8770 (5)	0.8446 (5)	0.9251 (5)	0.0989 (16)	
C51	0.8485 (7)	0.7476 (7)	0.9162 (6)	0.103 (2)	
H51	0.8955	0.7254	0.8679	0.123*	
C52	0.7534 (8)	0.6741 (6)	0.9729 (8)	0.109 (3)	
H52	0.7349	0.6046	0.9639	0.131*	
C53	0.6881 (6)	0.7093 (5)	1.0433 (7)	0.107 (3)	
H53	0.6222	0.6648	1.0856	0.128*	
C54	0.7233 (6)	0.8112 (6)	1.0487 (6)	0.0923 (19)	
H54	0.6799	0.8361	1.0971	0.111*	
N60	1.2999 (4)	0.5993 (4)	1.0408 (4)	0.0718 (11)	
N61	1.3853 (4)	0.6604 (4)	1.0600 (4)	0.0741 (12)	
C61	1.3687 (5)	0.7775 (5)	1.0313 (5)	0.0755 (15)	
H61	1.4294	0.8185	1.0462	0.091*	
C62	1.2676 (6)	0.8459 (5)	0.9802 (5)	0.0828 (17)	
H62	1.2607	0.9304	0.9593	0.099*	
C63	1.1803 (5)	0.7846 (5)	0.9622 (5)	0.0751 (14)	
H63	1.1088	0.8241	0.9299	0.090*	
C64	1.2007 (5)	0.6605 (5)	0.9935 (5)	0.0729 (14)	
H64	1.1411	0.6169	0.9805	0.088*	

### Atomic displacement parameters (Å^2^)

**Table:**

	*U*^11^	*U*^22^	*U*^33^	*U*^12^	*U*^13^	*U*^23^
Ni1	0.0371 (2)	0.0319 (2)	0.0395 (2)	0.00175 (16)	0.00078 (17)	−0.01283 (18)
N1	0.048 (2)	0.0464 (18)	0.0447 (19)	0.0053 (16)	−0.0025 (16)	−0.0168 (16)
C1	0.056 (3)	0.0323 (18)	0.043 (2)	−0.0050 (18)	0.0046 (19)	−0.0142 (17)
Se1	0.0648 (3)	0.0556 (3)	0.0712 (4)	0.0030 (2)	−0.0256 (3)	−0.0156 (3)
N2	0.0443 (19)	0.0453 (18)	0.048 (2)	0.0073 (15)	−0.0059 (16)	−0.0165 (16)
C2	0.044 (2)	0.0390 (19)	0.043 (2)	−0.0033 (17)	0.0035 (18)	−0.0166 (18)
Se2	0.0581 (3)	0.0605 (3)	0.0658 (3)	0.0073 (2)	−0.0216 (2)	−0.0175 (3)
N10	0.0373 (18)	0.0401 (16)	0.058 (2)	0.0012 (14)	−0.0016 (15)	−0.0221 (16)
N11	0.062 (2)	0.0395 (17)	0.061 (2)	−0.0087 (16)	0.0092 (18)	−0.0209 (17)
C11	0.062 (3)	0.047 (2)	0.083 (4)	−0.014 (2)	0.014 (3)	−0.024 (2)
C12	0.063 (3)	0.054 (3)	0.101 (4)	−0.010 (2)	0.000 (3)	−0.045 (3)
C13	0.080 (4)	0.079 (3)	0.089 (4)	−0.014 (3)	0.003 (3)	−0.059 (3)
C14	0.061 (3)	0.059 (3)	0.060 (3)	−0.011 (2)	0.003 (2)	−0.030 (2)
N20	0.0432 (18)	0.0324 (14)	0.0427 (18)	0.0033 (13)	−0.0005 (14)	−0.0140 (14)
N21	0.0462 (19)	0.0394 (16)	0.0478 (19)	0.0057 (14)	0.0030 (15)	−0.0154 (15)
C21	0.050 (3)	0.047 (2)	0.062 (3)	0.013 (2)	0.001 (2)	−0.023 (2)
C22	0.059 (3)	0.068 (3)	0.058 (3)	0.006 (2)	0.011 (2)	−0.028 (2)
C23	0.066 (3)	0.051 (2)	0.048 (3)	−0.001 (2)	0.009 (2)	−0.019 (2)
C24	0.052 (2)	0.042 (2)	0.048 (2)	0.0027 (18)	0.0011 (19)	−0.0186 (19)
N30	0.0446 (19)	0.0385 (16)	0.054 (2)	−0.0024 (14)	0.0021 (16)	−0.0213 (16)
N31	0.059 (2)	0.0398 (17)	0.063 (2)	−0.0069 (16)	0.0085 (18)	−0.0194 (17)
C31	0.065 (3)	0.043 (2)	0.086 (4)	−0.006 (2)	0.007 (3)	−0.026 (2)
C32	0.060 (3)	0.058 (3)	0.105 (4)	−0.014 (2)	0.003 (3)	−0.045 (3)
C33	0.073 (3)	0.084 (4)	0.088 (4)	−0.017 (3)	0.001 (3)	−0.060 (3)
C34	0.060 (3)	0.058 (3)	0.062 (3)	−0.012 (2)	0.000 (2)	−0.031 (2)
N40	0.0414 (18)	0.0371 (16)	0.0481 (19)	0.0000 (14)	0.0026 (15)	−0.0159 (15)
N41	0.047 (2)	0.0401 (16)	0.052 (2)	0.0061 (15)	0.0027 (16)	−0.0157 (15)
C41	0.057 (3)	0.058 (3)	0.065 (3)	0.009 (2)	0.006 (2)	−0.032 (2)
C42	0.057 (3)	0.073 (3)	0.064 (3)	0.002 (2)	0.016 (2)	−0.031 (3)
C43	0.068 (3)	0.058 (3)	0.046 (3)	−0.015 (2)	0.016 (2)	−0.017 (2)
C44	0.057 (3)	0.039 (2)	0.047 (2)	−0.0022 (18)	0.000 (2)	−0.0127 (18)
N50	0.084 (3)	0.067 (3)	0.116 (4)	−0.002 (3)	−0.010 (3)	−0.050 (3)
N51	0.088 (4)	0.075 (3)	0.115 (4)	0.010 (3)	0.007 (3)	−0.030 (3)
C51	0.119 (6)	0.105 (5)	0.100 (5)	0.046 (5)	−0.027 (4)	−0.062 (4)
C52	0.129 (6)	0.057 (3)	0.156 (7)	0.027 (4)	−0.089 (6)	−0.058 (4)
C53	0.066 (4)	0.059 (3)	0.156 (7)	−0.011 (3)	−0.016 (4)	−0.015 (4)
C54	0.095 (5)	0.088 (4)	0.094 (5)	0.012 (4)	0.000 (4)	−0.043 (4)
N60	0.080 (3)	0.049 (2)	0.078 (3)	−0.008 (2)	−0.006 (2)	−0.022 (2)
N61	0.072 (3)	0.061 (2)	0.075 (3)	−0.004 (2)	−0.005 (2)	−0.019 (2)
C61	0.082 (4)	0.072 (3)	0.077 (4)	−0.022 (3)	0.002 (3)	−0.037 (3)
C62	0.103 (5)	0.052 (3)	0.091 (4)	−0.002 (3)	0.018 (4)	−0.033 (3)
C63	0.065 (3)	0.075 (3)	0.080 (4)	0.009 (3)	0.007 (3)	−0.032 (3)
C64	0.075 (4)	0.074 (3)	0.072 (4)	−0.025 (3)	0.006 (3)	−0.035 (3)

### Geometric parameters (Å, º)

**Table:**

Ni1—N1	2.051 (4)	C32—C33	1.363 (8)
Ni1—N2	2.055 (4)	C32—H32	0.9300
Ni1—N10	2.154 (3)	C33—C34	1.391 (6)
Ni1—N20	2.129 (3)	C33—H33	0.9300
Ni1—N30	2.153 (3)	C34—H34	0.9300
Ni1—N40	2.124 (3)	N40—C44	1.317 (5)
N1—C1	1.151 (5)	N40—N41	1.339 (4)
C1—Se1	1.786 (5)	N41—C41	1.315 (5)
N2—C2	1.152 (5)	C41—C42	1.381 (7)
C2—Se2	1.794 (4)	C41—H41	0.9300
N10—C14	1.317 (6)	C42—C43	1.362 (6)
N10—N11	1.335 (5)	C42—H42	0.9300
N11—C11	1.326 (5)	C43—C44	1.387 (6)
C11—C12	1.371 (7)	C43—H43	0.9300
C11—H11	0.9300	C44—H44	0.9300
C12—C13	1.357 (8)	N50—N51	1.289 (7)
C12—H12	0.9300	N50—C54	1.301 (8)
C13—C14	1.398 (6)	N51—C51	1.280 (9)
C13—H13	0.9300	C51—C52	1.370 (11)
C14—H14	0.9300	C51—H51	0.9300
N20—C24	1.323 (5)	C52—C53	1.358 (11)
N20—N21	1.348 (4)	C52—H52	0.9300
N21—C21	1.317 (5)	C53—C54	1.336 (9)
C21—C22	1.384 (6)	C53—H53	0.9300
C21—H21	0.9300	C54—H54	0.9300
C22—C23	1.359 (6)	N60—N61	1.324 (6)
C22—H22	0.9300	N60—C64	1.322 (7)
C23—C24	1.386 (6)	N61—C61	1.296 (6)
C23—H23	0.9300	C61—C62	1.380 (8)
C24—H24	0.9300	C61—H61	0.9300
N30—C34	1.301 (5)	C62—C63	1.336 (8)
N30—N31	1.335 (5)	C62—H62	0.9300
N31—C31	1.334 (5)	C63—C64	1.376 (7)
C31—C32	1.356 (7)	C63—H63	0.9300
C31—H31	0.9300	C64—H64	0.9300
			
N1—Ni1—N2	179.88 (15)	N31—C31—H31	117.4
N1—Ni1—N40	90.53 (13)	C32—C31—H31	117.4
N2—Ni1—N40	89.57 (13)	C31—C32—C33	116.5 (4)
N1—Ni1—N20	90.85 (13)	C31—C32—H32	121.8
N2—Ni1—N20	89.05 (13)	C33—C32—H32	121.8
N40—Ni1—N20	178.62 (14)	C32—C33—C34	117.1 (5)
N1—Ni1—N30	91.64 (13)	C32—C33—H33	121.5
N2—Ni1—N30	88.30 (13)	C34—C33—H33	121.5
N40—Ni1—N30	92.71 (12)	N30—C34—C33	123.9 (5)
N20—Ni1—N30	87.31 (12)	N30—C34—H34	118.0
N1—Ni1—N10	88.17 (13)	C33—C34—H34	118.0
N2—Ni1—N10	91.89 (13)	C44—N40—N41	120.0 (3)
N40—Ni1—N10	88.07 (12)	C44—N40—Ni1	126.0 (3)
N20—Ni1—N10	91.92 (12)	N41—N40—Ni1	114.0 (2)
N30—Ni1—N10	179.20 (13)	C41—N41—N40	118.7 (3)
C1—N1—Ni1	164.0 (3)	N41—C41—C42	123.6 (4)
N1—C1—Se1	179.7 (4)	N41—C41—H41	118.2
C2—N2—Ni1	167.3 (4)	C42—C41—H41	118.2
N2—C2—Se2	178.9 (4)	C43—C42—C41	117.8 (4)
C14—N10—N11	120.1 (3)	C43—C42—H42	121.1
C14—N10—Ni1	122.3 (3)	C41—C42—H42	121.1
N11—N10—Ni1	117.5 (3)	C42—C43—C44	116.7 (4)
C11—N11—N10	118.0 (4)	C42—C43—H43	121.6
N11—C11—C12	124.6 (5)	C44—C43—H43	121.6
N11—C11—H11	117.7	N40—C44—C43	123.1 (4)
C12—C11—H11	117.7	N40—C44—H44	118.4
C13—C12—C11	117.2 (4)	C43—C44—H44	118.4
C13—C12—H12	121.4	N51—N50—C54	119.0 (5)
C11—C12—H12	121.4	C51—N51—N50	119.5 (6)
C12—C13—C14	117.1 (5)	N51—C51—C52	124.4 (7)
C12—C13—H13	121.4	N51—C51—H51	117.8
C14—C13—H13	121.4	C52—C51—H51	117.8
N10—C14—C13	122.9 (5)	C53—C52—C51	115.7 (5)
N10—C14—H14	118.5	C53—C52—H52	122.2
C13—C14—H14	118.5	C51—C52—H52	122.2
C24—N20—N21	120.4 (3)	C54—C53—C52	116.6 (6)
C24—N20—Ni1	124.8 (2)	C54—C53—H53	121.7
N21—N20—Ni1	114.6 (2)	C52—C53—H53	121.7
C21—N21—N20	118.4 (3)	N50—C54—C53	124.8 (7)
N21—C21—C22	123.7 (4)	N50—C54—H54	117.6
N21—C21—H21	118.1	C53—C54—H54	117.6
C22—C21—H21	118.1	N61—N60—C64	118.5 (4)
C23—C22—C21	117.4 (4)	C61—N61—N60	118.9 (5)
C23—C22—H22	121.3	N61—C61—C62	124.9 (5)
C21—C22—H22	121.3	N61—C61—H61	117.6
C22—C23—C24	117.9 (4)	C62—C61—H61	117.6
C22—C23—H23	121.1	C63—C62—C61	116.7 (5)
C24—C23—H23	121.1	C63—C62—H62	121.7
N20—C24—C23	122.1 (4)	C61—C62—H62	121.7
N20—C24—H24	118.9	C62—C63—C64	117.0 (5)
C23—C24—H24	118.9	C62—C63—H63	121.5
C34—N30—N31	119.5 (3)	C64—C63—H63	121.5
C34—N30—Ni1	121.9 (3)	N60—C64—C63	124.1 (5)
N31—N30—Ni1	118.6 (3)	N60—C64—H64	118.0
C31—N31—N30	117.9 (4)	C63—C64—H64	118.0
N31—C31—C32	125.1 (5)		
